# The m^6^A modification of LINC01133 suppresses ER^+^ breast cancer progression by modulating IGF2BP2 protein stability via a ubiquitination-dependent mechanism

**DOI:** 10.3389/fonc.2025.1608574

**Published:** 2025-06-26

**Authors:** Mai-dong Li, Ben-jie Shan, Lei Wang, Shuang Gao, Li Hao, Hai-yang Yu, Yue-yin Pan

**Affiliations:** ^1^ Cheeloo College of Medicine, Shandong University, Jinan, Shandong, China; ^2^ Department of Medical Oncology, The First Affiliated Hospital of University of Science and Technology of China, Division of Life Sciences and Medicine, University of Science and Technology of China, Hefei, Anhui, China; ^3^ Department of Orthopaedics, The First Affiliated Hospital of University of Science and Technology of China, Division of Life Sciences and Medicine, University of Science and Technology of China, Hefei, Anhui, China

**Keywords:** LINC01133, IGF2BP2, m^6^A, METTL3, heterogenous

## Abstract

**Background:**

Breast cancer is characterized as highly heterogenous and is a representative model to understand how molecular features of tumor biology determine therapeutic strategy. LINC01133 exhibits opposing expressing patterns across different breast cancer subtypes, yet its roles and mechanisms in ER^+^ breast cancer remain a loaded question.

**Methods:**

The expression of LINC01133 was initially assessed utilizing a public dataset TCGA and subsequently validated within clinical samples through RT-qPCR and *in situ* hybridization (ISH). To determine the role of LINC01133, various assays, including colony formation, Transwell, 5-ethynyl-2′-deoxyuridine (EdU) labeling, and mouse xenograft experiments, were performed. Additionally, RNA immunoprecipitation (RIP), RNA pull-down, mass spectrometry (MS), and RNA stability assays were conducted to elucidate its mechanisms.

**Results:**

LINC01133 was dramatically downregulated in ER^+^ breast cancer, which results in unfavorable prognosis. Functionally, LINC01133 inhibited migration and invasion *in vitro* and metastasis *in vivo* of ER^+^ breast cancer cells. Mechanistically, LINC01133 can directly interact with IGF2BP2 protein promoting its ubiquitination and degradation. The downregulation of LINC01133 was mediated by m^6^A modification, catalyzed by METTL3 and recognized by YTHDF2, causing half-life reduction and accelerated degradation of LINC01133.

**Conclusion:**

Our findings revealed the downregulation of LINC01133 in ER^+^ breast cancer and provided novel insight to the role of METTL3/YTHDF2/LINC01133/IGF2BP2 axis in ER^+^ breast cancer, which might offer a novel perspective in the design and development of novel anticancer drugs.

## 1 Introduction

In 2020, global statistics data indicated that breast cancer reached the highest rate of incidence among cancers, as 2.3 million individuals were diagnosed, representing 11.7% of all cancer cases. Additionally, it holds the position of the fifth primary factor contributing to cancer-related mortality worldwide accounting for approximately 685,000 deaths (1). Breast cancer is highly heterogenous, representing variable morphologic and biological characteristics, and responding differently to the same therapy strategy (2). According to the histological characteristics, breast cancer is clinically classified into three categories as follows: hormone receptor-positive (ER^+^), human epidermal growth factor receptor-2-overexpressing (HER2^+^), and triple-negative breast cancer (TNBC) (3). Understanding the concrete genetic alteration and molecular mechanisms of different breast cancer subtypes can make an accurate diagnosis and provide precise therapy strategy for breast cancer.

Advances in the extensive full-length cDNA sequencing uncovered a novel category of regulatory non-coding RNAs with transcripts >200 nucleotides and limited protein-coding potential, which was subsequently defined as long non-coding RNAs (lncRNAs) (4). Initially regarded as transcriptional noise, lncRNAs are now recognized for their roles in chromatin remodeling, RNA stabilization, and transcriptional regulation (5, 6). Dysregulated lncRNA expression is linked to cancer development, progression, and therapeutic resistance making them potential diagnostic makers and therapeutic target (7). Of the countless lincRNAs, LINC01133, located in chromosome 1q23.2 with a length of 1,154 nt, has caught our attention because it exhibits contrasting expression patterns in different subtypes of breast cancer, e.g., it was downregulated in breast cancer (8), while elevated in TNBC (9). Nevertheless, its expression and molecule mechanisms in ER^+^ breast cancer remain poorly understood.

Among multiple post-transcriptional modifications in eukaryote, it is widely believed that N^6^-methyladenosine (m^6^A) is the most dynamical and plentiful type sharing approximately 80% of mRNA modifications (10–13). The m^6^A modification is subtly mediated in a sophisticated network including various enzymes and reader proteins. The methylation process is catalyzed by the m^6^A methyltransferase complex, comprising METTL3, METTL14, and WTAP (14–16), while demethylation is mediated by m^6^A demethylases, including Fat mass obesity-associated protein (FTO) and Alk B homolog 5 (ALKBH5) (17). The m^6^A modification is decoded and recognized, and the message and signal downstream are processed by the following distinct classes of reader proteins: the YTH domain family (YTHDF1, YTHDF2, and YTHDF3) (18), YTH domain-containing proteins (YTHDC1 and YTHDC2), and IGF2BPs (IGF2BP1, IGF2BP2, and IGF2BP3). The reader proteins specifically interact with m^6^A-modified sites, thus influencing multiple points of RNA metabolism, including RNA half-life, nuclear export, splicing, and translation processes (19, 20). Based on these, dysregulated m^6^A modification is closely linked to cancer progression.

In this research, our objective is to elucidate the function and molecule mechanisms of LINC01133 in ER^+^ breast cancer.

## 2 Materials and methods

### 2.1 Patient tissue specimens

Patient tissue specimens were collected in The First Affiliated Hospital of USTC. Before collecting the tissues, written informed consent was acquired from volunteering patients. Additionally, ethical approval was granted from the Institutional Research Ethics Committee. All operations followed the guidelines of the ethics committee of The First Affiliated Hospital of USTC, as well as the Declaration of Helsinki (2008) of the World Medical Association and its subsequent amendments or equivalent ethical standards. The information of clinicopathological characteristics of ER^+^ breast cancer specimens are shown in **Supplementary Table S1**.

### 2.2 Cell lines and cell culture

The human breast cancer cell lines MCF-7 and T47D were obtained from FuHeng Cell Center (Shanghai, China), and DMEM medium (Gibco, California, USA) supplemented with 10% fetal bovine serum (FBS, Gibco, California, USA) and 1% penicillin/streptomycin antibiotic (Gibco, California, USA) were employed to culture the cells. All cell cultures were maintained at 37°C in a constant-humidified incubator containing 5% CO_2_.

### 2.3 Establishing stable cell lines

The LINC01133-overexpressing plasmid (LINC01133) and LINC01133-knockdown lentiviral plasmid (sh#1 and sh#2) were prepared by Umine Biotechnology Co., LTD (Guangzhou, China). Subsequently, we accomplished transfection of the plasmids using the Lipofectamine 3000 reagent (Invitrogen, Carlsbad, USA) following the instructions. The lentiviral plasmids were used to package the viral particles. Finally, we infected the cells with the virus and screened the stable cell lines using 1 μg/ml of puromycin (Gibco, California, USA) for 10 days.

### 2.4 RNA extraction and qRT-PCR assay

Total RNA extraction from tissues or cells was performed using TRIzol reagent (Invitrogen, Carlsbad, USA) following the instructions. PrimeScript™ RT reagent Kit (Takara, Kyoto, Japan) was applied to synthesize the cDNA. qRT‐PCR was completed using the SYBR Premix Ex Taq kit (Takara, Kyoto, Japan). We used GAPDH as the internal control to calculate mRNA levels. The RNA primers are as follows: LINC01133, forward, 5′-CCTAATCTCACCACAGCCTGG-3′, reverse, 5′-TCAGAGGCACTGATGTTGGG-3′; GAPDH, forward, 5′-GTCTCCTCTGACTTCAACAGCG-3′, reverse, 5′-ACCACCCTGTTGCTGTAGCCAA-3′.

### 2.5 *In situ* hybridization

ISH was performed in paraffin-embedded ER^+^ breast cancer samples to estimate LINC01133 expression according to the method previously described (21). The sequence of biotin-labeled LINC01133 probe is as follows: 5′-GGAGGTAAAGAGTAGAAGACAGTATCAAGAATCCAGAG-3′.

### 2.6 RNA stability assay

First, cells were cultivated using six-well plates for 24 h. Subsequently, 5 μg/ml of actinomycin D (Cat: HY-17559; Med Chem Express, Monmouth Junction, NJ, USA) was used to treat cells to inhibit gene transcription. RNA extraction was completed, and examined by RT-qPCR assay. Finally, RNA levels of different groups were measured at different time points. In addition, the levels of GAPDH mRNA also served as controls.

### 2.7 Mouse xenograft model

All animal experiments were priorly approved by the institutional animal use and care committee of The First Affiliated Hospital of USTC and conducted in line with the protocol established by this committee. Female 4-week-old BALB/c mice were obtained from Zhuhai BesTest Bio-Tech Co., Ltd. (Zhuhai, China), fed in a pathogen-free room at 22°C with 60% relative humidity, and subjected to a 12-h light/dark cycle.

For tumor growth analysis, 5 × 10^6^ cells were subcutaneously injected into the flank of each mouse. For metastatic potential analysis, every mouse tail vein was injected with 5 × 10^6^ cells. Tumor volumes were evaluated every 3 days following the equation (L × W^2^)/2, where L represents the longest diameter, and W indicates the shortest diameter perpendicular to L.

### 2.8 RIP, RNA pull-down, and mass spectrometry

RIP, RNA pull-down, and mass spectrometry were accomplished following the methods reported previously (8).

RIP assays were performed using the EZ-Magna RIP kit (Merck, Darmstadt, Germany) following the manufacturer’s protocol. Briefly, 2 × 10^7^ prostate cells were lysed in RIPA buffer (Beyotime, China) supplemented with protease and RNase inhibitors. Ten percent of the lysate was reserved as pre-immunoprecipitation input. Beads were washed three times with RIP buffer, resuspended in 500 μl of RIP buffer, and incubated with 5 μg of anti-GTF2F2 antibody (#MA5-44917, Thermo Fisher Scientific, Waltham, MA, USA) at 4°C for 8 h. After centrifugation at 3,000 rpm for 2 min to remove supernatants, 300 μl of cell lysate was added to the antibody-bound beads and incubated overnight at 4°C. IgG served as the negative control. Immunoprecipitated RNAs were subsequently eluted and quantified by qRT-PCR.

RNA pull‐down assay was performed following the protocol. Briefly, 1 × 10^7^ cells were lysed on ice for 10 min in 500 μl of polysome extraction buffer [100 mM KCl, 5 mM MgCl_2_, 5% NP-40, and 20 mM Tris-HCl (pH 7.4)]. The supernatant was collected and incubated with 1–2 μg of biotin-labeled probes at room temperature for 30 min to allow RNA–protein complex formation. Pre-treated streptavidin magnetic beads (BioLabs, Massachusetts, USA) were then added to the reaction mixture and incubated at room temperature for an additional 30 min. Following six washes with RNA washing buffer, the precipitated RNA–protein complexes were analyzed by Western blotting. Bound proteins were subsequently eluted and analyzed using mass spectrometry (LTQ Orbitrap Velos Pro, Thermo Scientific, Waltham, MA, USA).

### 2.9 Western blotting assay

Total protein extraction from cells or tissues was accomplished using RIPA (Sigma-Aldrich, St. Louis, MO, USA). We used the Pierce™ BCA Protein Assay Kit (Thermo Fisher Scientific, Waltham, MA, USA) to evaluate the concentration of protein. To separate the protein, 10% SDS-PAGE gel (BeyoGel™ Plus PAGE, Beyotime, Shanghai, China) was employed. The process of Western blotting assay was executed following an established protocol (22). In this research, the primary antibodies employed were anti-IGF2BP2 (Cat: MA5-44917; Thermo Fisher Scientific, Waltham, MA, USA) and anti-GAPDH (Cat: 437000, Invitrogen, Carlsbad, USA). GAPDH was employed as an internal loading control, which can normalize and verify equal protein loading among all experimental samples.

### 2.10 Cellular functional assays

To research the functions of LINC01133 on breast cancer *in vitro*, we performed some cellular functional assays including colony formation, CCK-8, EdU, and Transwell following previously established protocols (23).

### 2.11 Statistical analysis

All experiments were repeated more than three times. We accomplished the statistical analysis via SPSS 23.0 software (IBM, USA). The mean ± standard deviation was employed to display the statistical analysis results. Differences between paired tissues were analyzed using Student’s paired t-test, whereas comparisons among more than two groups were examined via variance (ANOVA), along with Dunnett’s test. A value of p below 0.05 was considered statistically significant.

## 3 Results

### 3.1 LINC01133 is significantly downregulated in ER^+^ breast cancer, which results in unfavorable survival

**Figure 1:**
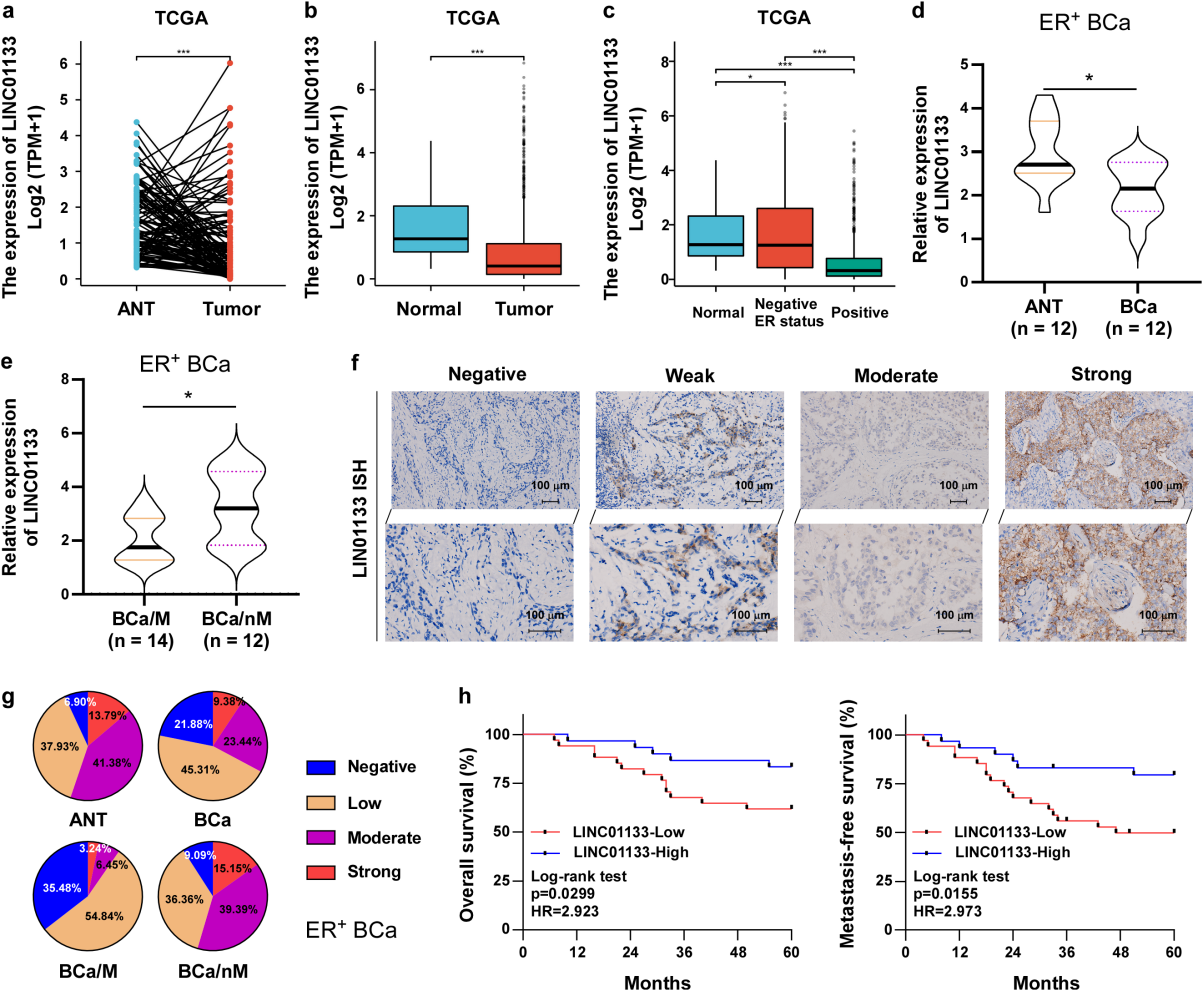
LINC01133 was significantly downregulated in ER+ breast cancer, which resulted in unfavorable survival. **(a)** LINC01133 was significantly decreased in tumor tissues compared to matched adjacent normal breast tissues (ANT) based on data sourced from TCGA. **(b)** LINC01133 expression in breast cancer tissues was much lower than those in normal breast tissues based on data derived from TCGA. **(c)** LINC01133 was progressively decreased from normal breast tissues, ER^−^ breast cancer tissues, to ER^+^ breast cancer tissues using data sourced from TCGA. **(d)** RT-qPCR uncovered that LINC01133 is robustly downregulated in ER^+^ breast cancer (BCa, n = 12) relative to matched ANT (n = 12). **(e)** LINC01133 was dramatically abnormally downregulated in ER^+^ breast cancer with metastasis (BCa/M, n = 14) compared with ER^+^ breast cancer without metastasis (BCa/nM, n = 12). **(f)** ISH assay of LINC01133 in ER^+^ breast cancer tissues. **(g)** Statistical chart of ISH levels in different ER^+^ breast cancers. **(h)** The overall survival and metastasis-free survival of ER^+^ breast cancer patients with low LINC01133 was shorter than that with high LINC01133. *p < 0.5; ***p < 0.001.

To assess LINC01133 levels in breast cancer, we initially analyzed the data derived from The Cancer Genome Atlas (TCGA) database. As exhibited in **Figures 1a–c**, LINC01133 was significantly downregulated in tumor tissues compared to their matched adjacent normal breast tissue (ANT; **Figure 1a**). In addition, LINC01133 levels in breast cancer tissues are much lower than those in normal breast tissues (**Figure 1b**). Later, we compared LINC01133 expression in ER^−^ and ER^+^ breast cancers. The results revealed a progressive decrease in LINC01133 levels from normal breast tissues, ER^−^ breast cancer tissues, to ER^+^ breast cancer tissues (**Figure 1c**). Given the notable reduction in ER^+^ breast cancer tissues, we then investigated its expression and role in this subtype. Likewise, real-time PCR assay uncovered that LINC01133 was robustly decreased in ER^+^ breast cancer (BCa, n = 12) relative to corresponding adjacent normal breast tissues (ANT, n = 12) (**Figure 1d**). To test whether LINC01133 is linked to metastasis of ER^+^ breast cancer, its expression was examined in 14 cases of ER^+^ breast cancer with metastasis (BCa/M, n = 14) and 12 cases of ER^+^ breast cancer without metastasis (BCa/nM, n = 12). The results revealed that LINC01133 was significantly abnormally downregulated in BCa/M compared with BCa/nM (**Figure 1e**). Consistently, ISH assay illustrated a significant decrease in LINC01133 in ER^+^ breast cancer specimens compared to ANT, and in BCa/M compared to BCa/nM, respectively (**Figures 1f, g**). Additionally, low LINC01133 predicted shorter overall survival and metastasis-free survival compared to those with high LINC01133 in ER^+^ breast cancer patients (**Figure 1h**).

Collectively, these findings demonstrated that a notable reduction in LINC01133 might play an indispensable function in the breast cancer progression, especially in ER^+^ breast cancer.

### 3.2 LINC01133 suppresses ER^+^ breast cancer cell proliferation and metastasis

**Figure 2:**
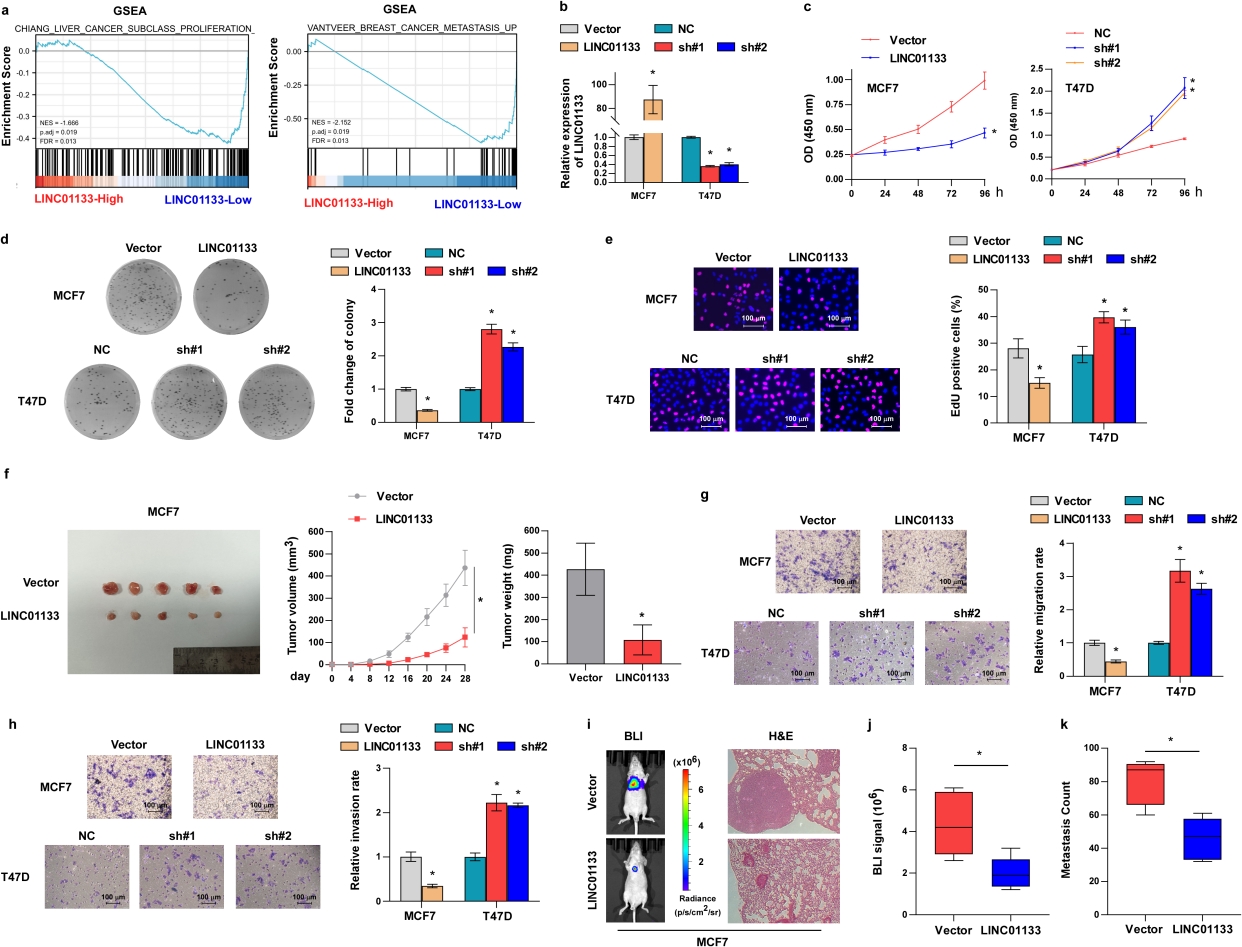
LINC01133 suppresses ER^+^ breast cancer cell proliferation and metastasis. **(a)** GSEA analysis revealed that proliferation and metastasis traits were abundant in the LINC01133-low ER^+^ breast cancer subgroup. **(b)** Levels of LINC01133 in stable cell lines. **(c)** CCK8 assay of different stable cell lines. **(d)** Representative images and statistical chart of colony formation formed by MCF-7 and T47D cells. **(e)** Representative images and statistical chart of EdU assay formed by MCF-7 and T47D cells. **(f)** Subcutaneous tumor of BALB/c nude mice formed by MCF-7 cells and corresponding tumor volume and tumor weight. **(g)** Representative migration images and statistical chart formed by MCF-7 and T47D cells. **(h)** Representative invasive images and statistical chart formed by MCF-7 and T47D cells. **(i)** Representative images of bioluminescence imaging and H&E staining of lung tissues of mice. **(j)** Statistical chart of bioluminescence signal in different mouse groups. **(k)** Metastasis tumor count of lungs in different mouse groups. *p < 0.5.

To elucidate the roles of LINC01133 in ER^+^ breast cancer, researchers performed a gene set enrichment analysis (GSEA) on ER^+^ breast cancer samples sourced from TCGA. GSEA analysis revealed a notable trend of proliferation traits focused on the subset of ER^+^ breast cancer exhibiting low levels of LINC01133 (**Figure 2a**) suggesting that LINC01133 might mediate the proliferation of ER^+^ breast cancer. Subsequently, a stable cell line upregulating LINC01133 was constructed using MCF-7 cells (**Figure 2b**). Conversely, a stable cell line exhibiting low levels of LINC01133 was established using T47D cells (**Figure 2b**). Functional experiments of cell proliferation, including CCK8 (**Figure 2c**), colony formation (**Figure 2d**), and EdU (**Figure 2e**), demonstrated that inhibition of LINC01133 notably promoted, while overexpression of LINC01133 suppressed, the proliferation of ER^+^ breast cancer cells. Additionally, we subcutaneously injected luciferase-labeled MCF-7 cells with or without LINC01133 overexpression into BALB/c nude mice. All mice were euthanized at 28 days. Downregulation of LINC01133 significantly inhibited tumor growth, as indicated by the decreased volume and weight (**Figure 2f**).

The GSEA assay indicated that LINC01133 may also be involved in tumor metastasis (**Figure 2a**), which was initially examined *in vitro*. Transwell assay demonstrated that the downregulation of LINC01133 importantly strengthened the migration and invasion capabilities of ER^+^ breast cancer cells, whereas LINC01133 upregulation substantially reduced these abilities (**Figures 2g, h**). Subsequently, the function of LINC01133 in cancer metastasis was assessed *in vivo*. Luciferase-labeled MCF-7 cells were introduced to the tail vein of the mice, with or without LINC01133 overexpression. Notably, bioluminescence imaging and H&E staining demonstrated that the overexpression of LINC01133 effectively hindered lung metastasis (**Figures 2i–k**).

Collectively, the abovementioned findings uncovered that LINC01133 regulates the aggressiveness of ER^+^ breast cancer.

### 3.3 LINC01133 interacts with IGF2BP2 protein to regulate its stability in a ubiquitination-dependent manner

**Figure 3:**
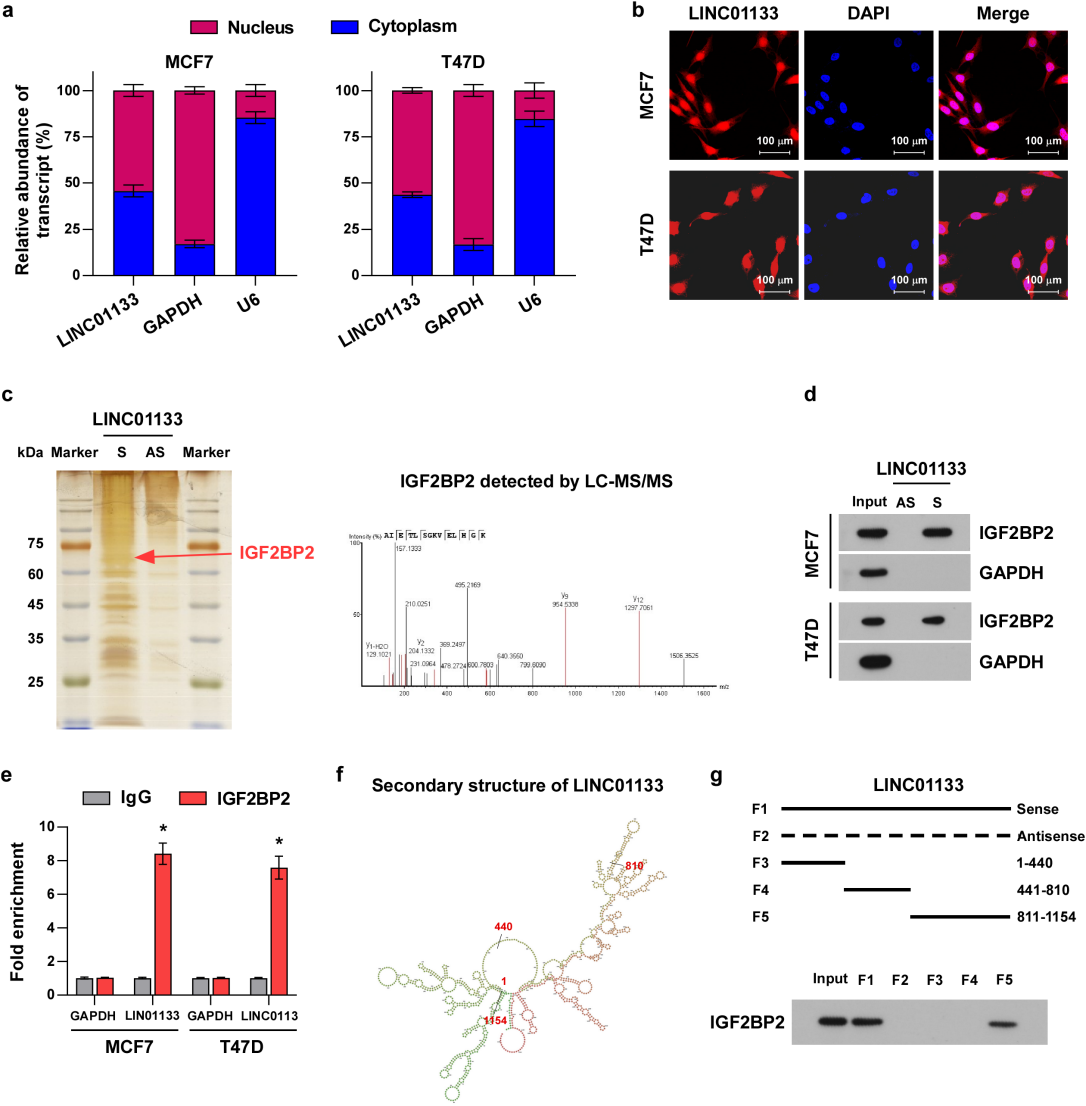
LINC01133 can directly interact with IGF2BP2. **(a)** LINC01133 expression in different subcellular fractionations. **(b)** RNA-FISH of LINC01133 in MCF-7 and T47D. **(c)** Band and mass spectrometry in silver staining pulled by biotinylated LINC01133. **(d)** Pull-down assay showed that LINC01133 can directly interact with IGF2BP2. **(e)** RIP assay demonstrated that LINC01133 interacted with IGF2BP2. **(f)** The secondary structure of LINC01133 predicted using the online tool RNAfold. **(g)** Pull-down experiments demonstrated that the F5 fragment (811–1,154 nt) is crucial for mediating the association between LINC01133 and IGF2BP2. *p < 0.5.

Since the localization of lncRNA is critical to their underlying mechanisms (24), we detected the localization of LINC01133 applying subcellular fractionation and RNA-FISH assays. These assays showed that LINC0113 is located in both the nucleus and cytoplasm (**Figures 3a, b**). LncRNAs fulfill multiple functions by directly interacting with their targeting proteins (25). To identify the interaction proteins of LINC01133, the possible interaction proteins were pulled using biotinylated probes of LINC01133, and the different band in silver staining was identified by mass spectrometry. Mass spectrometry analysis demonstrated a potential direct interaction between IGF2BP2 and LINC01133 (**Figure 3c**). This interaction was subsequently validated through independent experiments, including an RNA pull-down assay (**Figure 3d**) and RIP experiments (**Figure 3e**). To further clarify the specific fragments of LINC01133 interacting with IGF2BP2, we predicted the secondary structure of LINC01133 using an online tool RNAfold (**Figure 3f**). Based on the secondary structure analysis of LINC01133, we designed and constructed three deletion mutants while preserving their essential RNA hairpin structures. RNA pull-down experiments demonstrated that the F5 fragment (spanning nucleotides 811–1,154) is crucial for mediating the association between LINC01133 and IGF2BP2 (**Figure 3g**).

**Figure 4:**
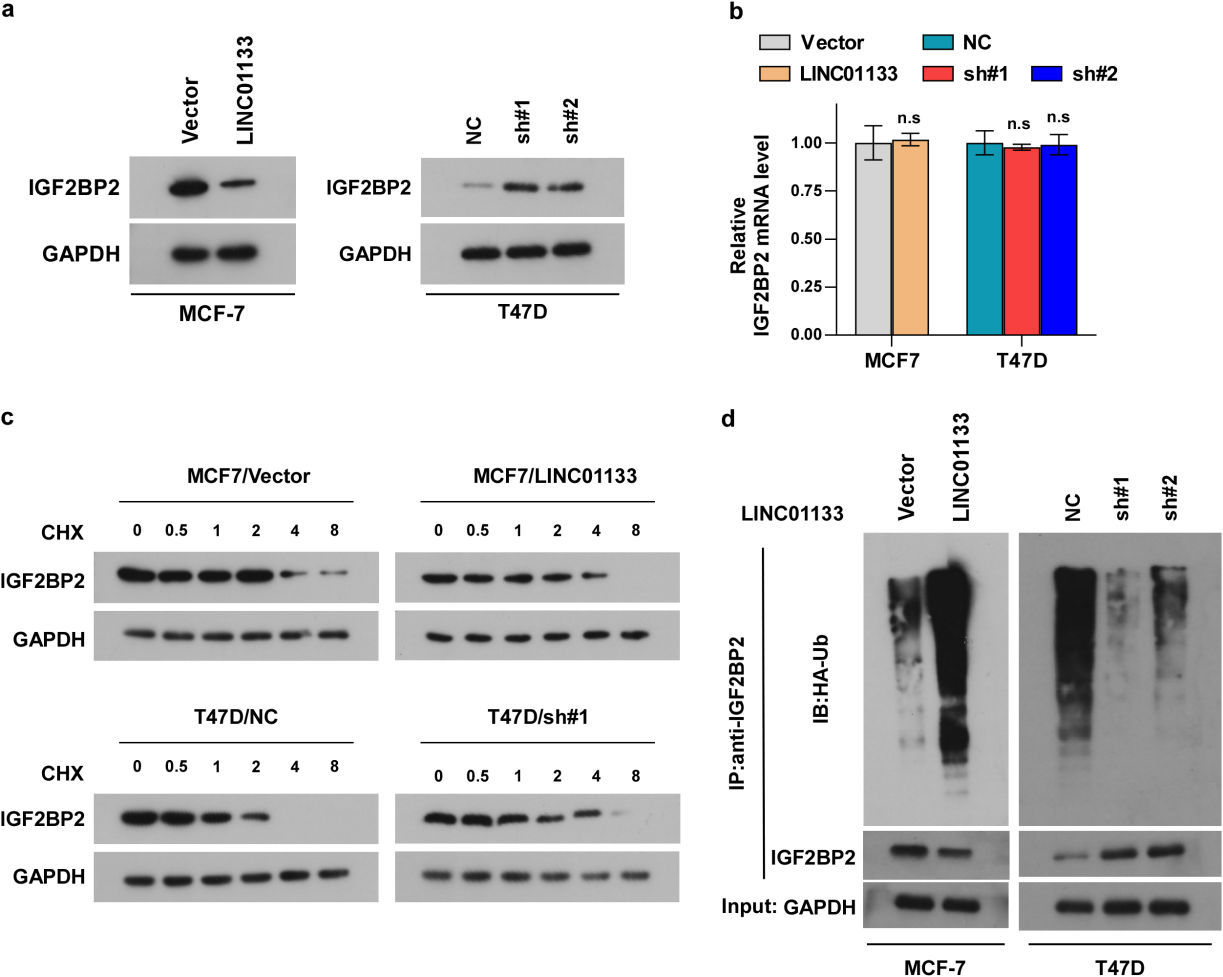
LINC01133 modulated IGF2BP2 stability via a ubiquitination-dependent manner. **(a)** Representative band of IGF2BP2 in different cell lines. **(b)** RT-qPCR assay demonstrated that either increasing or decreasing LINC01133 hardly influences the RNA levels of IGF2BP2. **(c)** Western blotting assay demonstrated that LINC01133 overexpression sharply promoted the degradation level of IGF2BP2, while downregulation of LINC01133 had the opposite effect. **(d)** Ubiquitination assay demonstrated that overexpression of LINC01133 dramatically promotes the ubiquitination of IGF2BP2, while downregulation of LINC01133 had the opposite effect. n.s., no significance.

To investigate whether the interaction of LINC01133 with IGF2BP2 influences the protein level of IGF2BP2, several experiments were performed. The results clarified that overexpression of LINC01133 inhibited, while LINC01133 knockdown increased, the protein level of IGF2BP2 (**Figure 4a**). However, either increasing or decreasing LINC01133 hardly influences the RNA levels of IGF2BP2 (**Figure 4b**) inferring that LINC01133 might participate in the protein, rather than RNA, mediation process of IGF2BP2. Thus, we proposed a hypothesis that LINC01133 might regulate the protein stability of IGF2BP2. Then, we tested our hypothesis. We treated the cells using the proteasome inhibitor MG132 (10 µM), and detected the ubiquitination level of IGF2BP2, which reflects its degradation level by the 26S proteasome. As illustrated in **Figures 4c, d**, overexpression of LINC01133 sharply promoted the ubiquitination and degradation levels of IGF2BP2, while downregulation of LINC01133 had the opposite effect. These findings demonstrated that LINC01133 modulates the IGF2BP2 stability through a ubiquitination-dependent mechanism. However, knockdown of IGF2BP2 does not affect LINC01133 expression levels (**Supplementary Figure S1a**), RNA stability (**Supplementary Figures S1b, c**), or subcellular localization (**Supplementary Figure S1d**).

**Figure 5:**
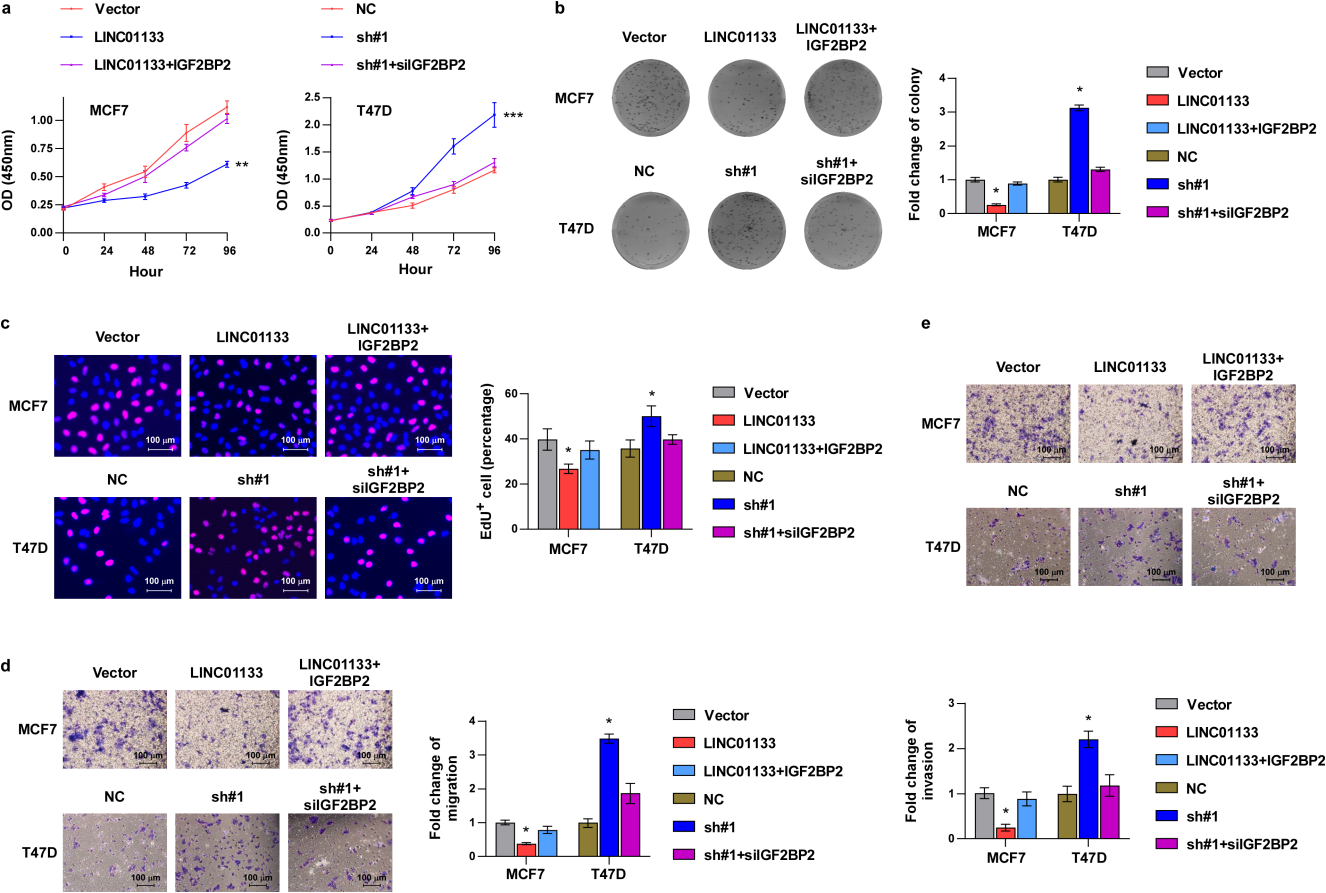
IGF2BP2 can partially reverse the suppressive effects induced by LINC01133 upregulation in ER^+^ breast cancer. **(a)** CCK8 assay of different cell lines. **(b)** Colony formation assay of different cell lines. **(c)** EdU labeling assay of different cell lines. **(d)** Migration assay of different cell lines. **(e)** Invasion assay of different cell lines. *p < 0.5. Collectively, the above findings elucidated that LINC01133 interacts with IGF2BP2 and enhances its ubiquitination degradation, and IGF2BP2 is essential for LINC01133 to modulate the progressive ability of ER^+^ breast cancer.

To further evaluate the influence of IGF2BP2 on LINC01133-induced proliferation and metastasis of ER^+^ breast cancer, IGF2BP2 was transiently transfected into LINC01133-upregulated cells, and siRNA targeting IGF2BP2 was transiently transfected into LINC01133-downregulated cells. CCK8 (**Figure 5a**), colony formation (**Figure 5b**), EdU (**Figure 5c**), migration (**Figure 5d**), and invasion assays (**Figure 5e**) showed that IGF2BP2 could partially rescue the suppressive effects induced by LINC01133 upregulation. Conversely, IGF2BP2 silencing could partially attenuate the promoting effects resulting from LINC01133 downregulation on proliferation, migration, and invasion of ER^+^ breast cancer cells, respectively.

### 3.4 m^6^A modification contributes to the downregulation of LINC01133 in ER^+^ breast cancer

**Figure 6:**
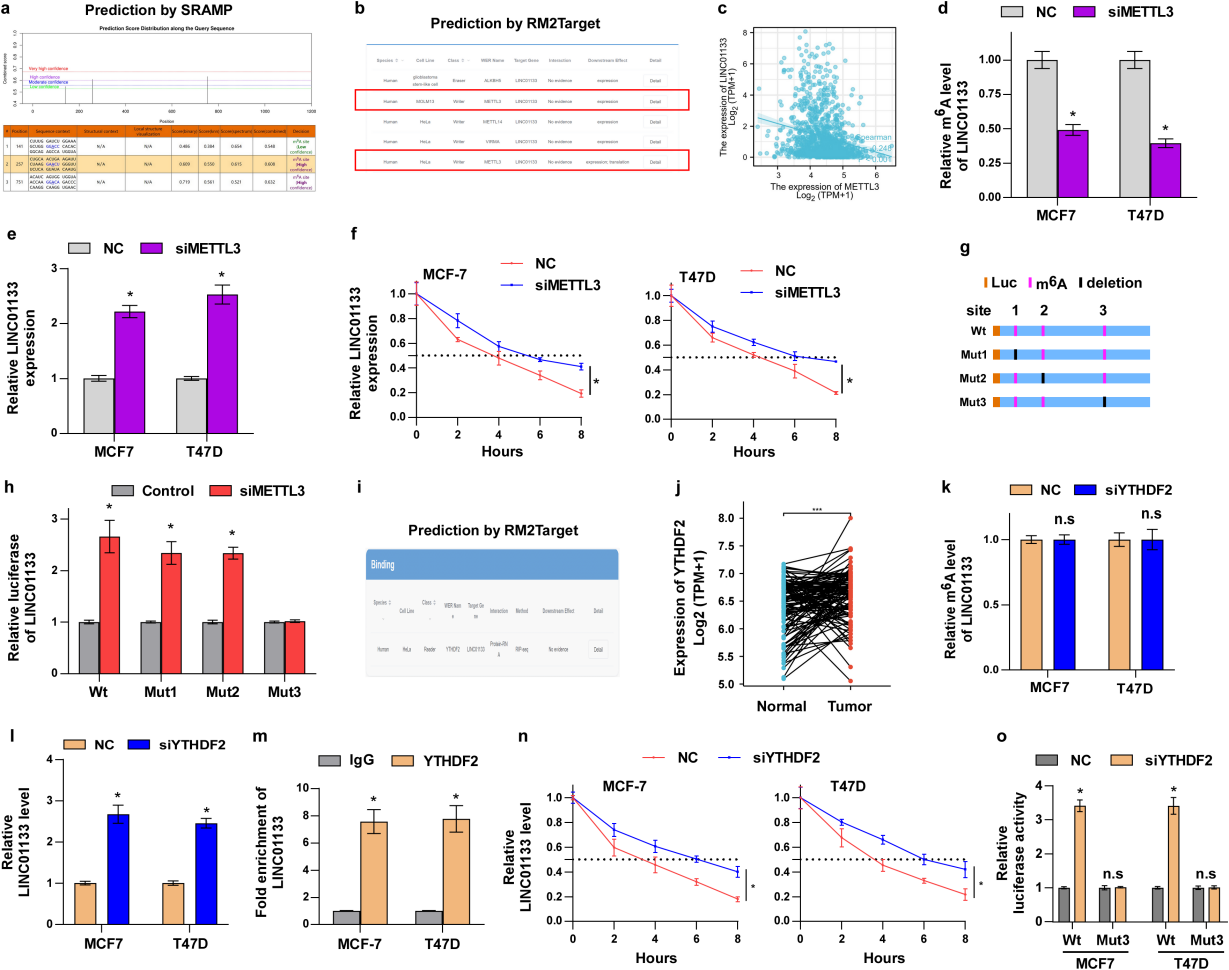
m^6^A modification contributes to the downregulation of LINC01133 in ER^+^ breast cancer. **(a)** The possible m^6^A modification site of LINC01133 predicted by the online tool SRAMP. **(b)** The online tool RM2Target predicted that METTL3 might mediate the m^6^A modification of LINC01133. **(c)** Data sourced from TCGA demonstrated that LINC01133 is negatively correlated with METTL3 level. **(d)** Relative m^6^A levels of LINC01133 in different cell lines. **(e)** Relative LINC01133 expression in different cell lines. **(f)** RNA stability assay of LINC01133 in MCF-7 and T47D cells. **(g)** Schematic diagram of the deleted potential m^6^A site of LINC01133. **(h)** Luciferase reporter assay demonstrated the activity of wild-type and mutant LINC01133 reporter in the indicated cells. **(i)** The online tool RM2Target predicted that YTHDF2 might co-precipitate with LINC01133. **(j)** Data sourced from TCGA demonstrated that YTHDF2 significantly elevates in ER^+^ breast cancer tissues relative to normal breast tissues. **(k)** Relative m^6^A levels of LINC01133 in different cell lines. **(l)** Relative LINC01133 expression in different cell lines. **(m)** RIP assay demonstrated that YTHDF2 co-precipitated with LINC01133. **(n)** RNA stability assay of LINC01133 in MCF-7 and T47D cells. **(o)** Luciferase reporter experiment demonstrated the activity of wild-type and mutant LINC01133 reporter in the indicated cells. *p < 0.5; n.s., no significance.

We subsequently researched the underlying mechanisms that lead to the decline of LINC01133 in ER^+^ breast cancer. Since m^6^A is the most widely reported RNA modification (26–29), we predicted whether the m^6^A modification site is located on the LINC01133 transcript using the online tool SRAMP. The prediction results showed that three sites on the LINC01133 transcript might be modified by m^6^A (**Figure 6a**). We further explored which writer is involved in the m^6^A modification of LINC01133 using the online tool RM2Target. The results showed that METTL3 might modulate the m^6^A modification of LINC01133 (**Figure 6b**). Analysis of data from TCGA showed that the LINC01133 level was negatively correlated with the METTL3 level (**Figure 6c**) inferring that METTL3-mediated m^6^A modification of LINC01133 might decrease its level. We then tested our hypothesis and found that downregulation of METTL3 could significantly inhibit the m^6^A level of LINC01133 (**Figure 6d**) and promote the LINC01133 expression (**Figure 6e**). Moreover, we examined the influence of METTL3 downregulation on LINC01133 stability, and a significant increase was observed in the RNA half-life of LINC01133 (**Figure 6f**) inferring that METTL3 regulated the stability of LINC01133 via m^6^A modification. Finally, the online tool SRAMP predicted that m^6^A modification might be located at three sites, and we mutated the three sites, respectively, to investigate which site is responsible for the m^6^A-induced RNA stability (**Figure 6g**). Luciferase reporter assay showed that site 3 was essential for METTL3-mediated m^6^A modification of LINC01133 (**Figure 6h**).

Since m^6^A modification requires a reader to recognize the m^6^A base, we next examined which reader protein is responsible for this function. Through the online tool RM2Target, we found that YTHDF2, an m^6^A reader, might interact with LINC01133 (**Figure 6i**). Moreover, data from TCGA also showed that YTHDF2 was significantly increased in ER^+^ breast cancer tissues relative to ANT (**Figure 6j**). Further assays indicated that downregulation of YTHDF2 hardly influences the m^6^A modification level of LINC01133 (**Figure 6k**), but significantly increased the RNA level of LINC01133 (**Figure 6l**). In addition, RIP assay showed that YTHDF2 was co-precipitated with LINC01133 (**Figure 6m**). These results suggested that YTHDF2 might regulate LINC01133 expression through m^6^A modification. To verify this hypothesis, we investigated the concrete molecular mechanisms of YTHDF2 on the regulation of LINC01133. Our assay showed that YTHDF2 knockdown significantly increased the stability of LINC01133 (**Figure 6n**). Further assays indicated that YTHDF2 regulated the LINC01133 level through recognizing m^6^A modification at site 3 (**Figure 6o**). Especially, as shown in **Supplementary Figure S1e**, we found that YTHDF2 is the only m^6^A reader binding to LINC01133 in ER^+^ breast cancer cells. Additionally, as shown in **Supplementary Figure S1f**, Western blotting analysis revealed a negative correlation between the protein levels of METTL3/YTHDF2 and LINC01133 expression using ER^+^ breast cancer tissues consistent with our mRNA data. These results further strengthen the biological relevance of the interaction between the m^6^A machinery and LINC01133 in breast cancer.

Collectively, our findings showed that METTL3 and YTHDF2 regulates the m^6^A level of LINC01133 and that m^6^A modification plays a critical function in modulating LINC01133 stability.

## 4 Discussion

Breast cancer serves as a paradigmatic model to understand how the molecular features of tumor biology direct therapeutic decision making (30), thereby underscoring the critical roles of molecular testing in improving diagnosis and patient survival outcomes. Therefore, it is essential to identify and investigate the underlying mechanisms of special genes in different subtypes of breast cancer. Several studies have been reported about LINC01133. Song et al. demonstrated that a notable reduction trend in LINC01133 was found in breast cancer, which resulted in an unfavorable prognosis and an aggressive phenotype (8). Another study also documented that LINC01133 was decreased in luminal breast cancer (31). Nevertheless, Tu et al. found that LINC01133 was a PI3K-regulated lncRNA and exerted pro-tumorigenic roles in triple-negative breast cancer (9). The two diametrically opposed modes of level and function of LINC01133 in different studies suggest that further investigation into the expression and function of LINC01133 across different subtypes of breast cancer is imperative. Then, we examined the expression profile and function roles of LINC01133 in ER^+^ breast cancer, because this subtype is the most popular form, comprising 60%–70% of all breast cancer cases (32, 33). Interestingly, in agreement with the findings reported by Song et al., we clarified that LINC01133 is dramatically downregulated in breast cancer, with an even more pronounced reduction observed in ER^+^ breast cancer subtypes. Subsequent investigations showed that LINC01133 was inversely associated with the survival of patients with ER^+^ breast cancer. Additionally, both *in vitro* and *in vivo* experiments supported that LINC01133 can weaken the progressive ability of ER^+^ breast cancer.

Investigation into the mechanisms revealed that LINC01133 has the ability to directly bind to the protein IGF2BP2 to regulate its stability. IGF2BP2 belongs to an RNA-binding protein that can directly bind to the 5′ untranslated region (UTR) of the mRNA for insulin-like growth factor (IGF2), thus influencing its translation and plays various physiological and pathological mechanisms (34). Previous studies showed that IGF2BP2 is a type 2 diabetes-associated gene. In recent years, increasing studies have demonstrated that IGF2BP2 is correlated with poor prognosis of multiple cancers. For instance, upregulation of IGF2BP2 results in shorter survival and poorer prognosis for patients with breast cancer (18), esophageal carcinoma (35), and hepatocellular carcinoma (36). Our research elucidated that IGF2BP2 could partly recuse the suppressing effect of LINC01133 on ER^+^ breast cancer, which inferred that IGF2BP2 is also beneficial to the aggressive phenotype of the ER^+^ breast cancer. These results align with the aforementioned studies. TRIM15 has been reported to ubiquitinate IGF2BP2 to enhance the function of phase separation and the maintenance of mRNA stability of TLR4 (37). Biao et al. found that TRIM21 promotes the degradation of IGF2BP2 via the K48–ubiquitin–lysosome pathway (38). We also validated the roles of TRIM21 and TRIM15 in regulating IGF2BP2 in breast cancer, but as shown in **Supplementary Figure S1g**, we did not detect any interaction between TRIM21/15 and IGF2BP2, or LINC01133. Future studies will validate whether LINC01133 recruits these (or other) E3 ligases to mediate IGF2BP2 ubiquitination in ER^+^ breast cancer.

Ubiquitination, standing as a crucial post-translational modification for protein, is widely observed in eukaryotes. It plays an indispensable function in mediating protein homeostasis and a wide array of cellular processes (39, 40). Moreover, lncRNAs can bind to proteins to regulate their ability in a ubiquitination-dependent manner (41–44). LncRNA GClnc1 facilitated the progression of osteosarcoma by stabilizing NONO through inhibiting FBXW7-mediated ubiquitination (44). In gastric cancer, LINC02139 modulated cell proliferation and apoptosis by interacting with and stabilizing XIAP through blocking its ubiquitination (43). HNF4A-AS1 suppressed hepatocellular carcinoma progression via enhancing PCBP2 degradation through ubiquitin and destabilizing ARG2 mRNA (42). In this study, we found that LINC01133 interacted with IGF2BP2 to inhibit its degradation mediated by ubiquitination, which promoted the deterioration of ER^+^ breast cancer.

Aberrant m^6^A modifications are closely correlated with the progression of cancer. Efforts to develop m^6^A-targeted therapeutics have employed both conventional medicine-based strategies and advanced modern drug development platforms (45). m^6^A modification represents a key mechanism underlying RNA expression dysregulation. Lang et al. found that lncRNA PCAT6 was modified by m^6^A in a METTL3-dependent manner, which promoted bone metastasis through IGF2BP2/IGF1R axis in prostate cancer (46). Li et al. elucidated that m^6^A methylation enhanced the levels of NUTM2B-AS1 by stabilizing its transcript, and the m^6^A-modified NUTM2B-AS1 facilitated hepatocellular carcinoma cell proliferation and stemness by epigenetically upregulating BMPR1A expression (47). In this study, m^6^A modification in LINC01133 was upregulated, which was recognized by YTHDF2, leading to the degradation of the LINC01133 transcript. Our study extends the significance of m^6^A modification in ER^+^ breast cancer. Especially, early growth response 1 (EGR1) serves as a member of the EGR family, and exerts its effects by promoting or inhibiting the transcription of its downstream genes. Deguan Lv et al. found that EGR1 can bind to the METTL3 promoter to directly regulate the expression of METTL3 in glioblastoma stem cells (48). Wang et al. showed that EGR1 servers as a transcription factor for METTL3 to promote METTL3 transcription in osteoporosis (49). Liao et al. showed that m^6^A reader YTHDF2 recognizes the m6A modification of EGR1 subsequently inducing their RNA degradation (50). Our qPCR data demonstrate that knockdown of EGR1 significantly reduces METTL3 expression while upregulating LINC01133 levels in ER^+^ breast cancer cells. Importantly, YTHDF2 expression remains unaffected by EGR1 depletion (**Supplementary Figure S1h**). These results suggest that EGR1 may regulate LINC01133 expression through METTL3-mediated m6A modification rather than through YTHDF2.

## 5 Conclusion

In conclusion, our findings clarified that LINC01133 is significantly downregulated in ER^+^ breast cancer. Functionally, LINC01133 inhibited the metastatic ability of ER^+^ breast cancer via mediating the IGF2BP2 stability via a ubiquitination-dependent manner. The downregulation of LINC01133 was mediated by m^6^A modification, which reduced its half-life and accelerates its degradation. The m^6^A site in LINC01133 transcript was catalyzed by METTL3 and recognized by YTHDF2. Investigating the distinct expression patterns of LINC01133 across various breast cancer subtypes highlights the importance of elucidating its precise expression profiles and underlying mechanisms in each subtype. Such insights could function in directing the design of the novel anticancer therapeutics.

## Data Availability

The raw data supporting the conclusions of this article will be made available by the authors, without undue reservation.

## Supplementary material

Supplementary Figure 1
**(a)** Knockdown of IGF2BP2 does not affect LINC01133 expression levels in ER+ breast cancer cells. **(b, c)** Knockdown of IGF2BP2 does not affect the RNA stability of LINC01133 in ER^+^ breast cancer cells. **(d)** Knockdown of IGF2BP2 does not affect subcellular location of LINC01133 in ER^+^ breast cancer cells. **(e)** IGF2BP1/3 cannot bind to LINC01133 in ER^+^ breast cancer cells. **(f)** A negative correlation exists between the protein levels of METTL3/YTHDF2 and LINC01133 expression using ER^+^ breast cancer tissues. **(g)** TRIM21/15 cannot interact with IGF2BP2 and LINC01133 in ER^+^ breast cancer cells. **(h)** Knockdown of EGR1 significantly reduces METTL3 expression while upregulating LINC01133 levels in ER^+^ breast cancer cells, while YTHDF2 remains unaffected by EGR1 depletion.
