# Supplementary figures and images for: The m6A modification of LINC01133 suppresses ER+ breast cancer progression by modulating IGF2BP2 protein stability via a ubiquitination-dependent mechanism
Source: Front Oncol. 2025 Jun 26;15:1608574. doi: 10.3389/fonc.2025.1608574 (PMC12241053; doi:10.3389/fonc.2025.1608574)

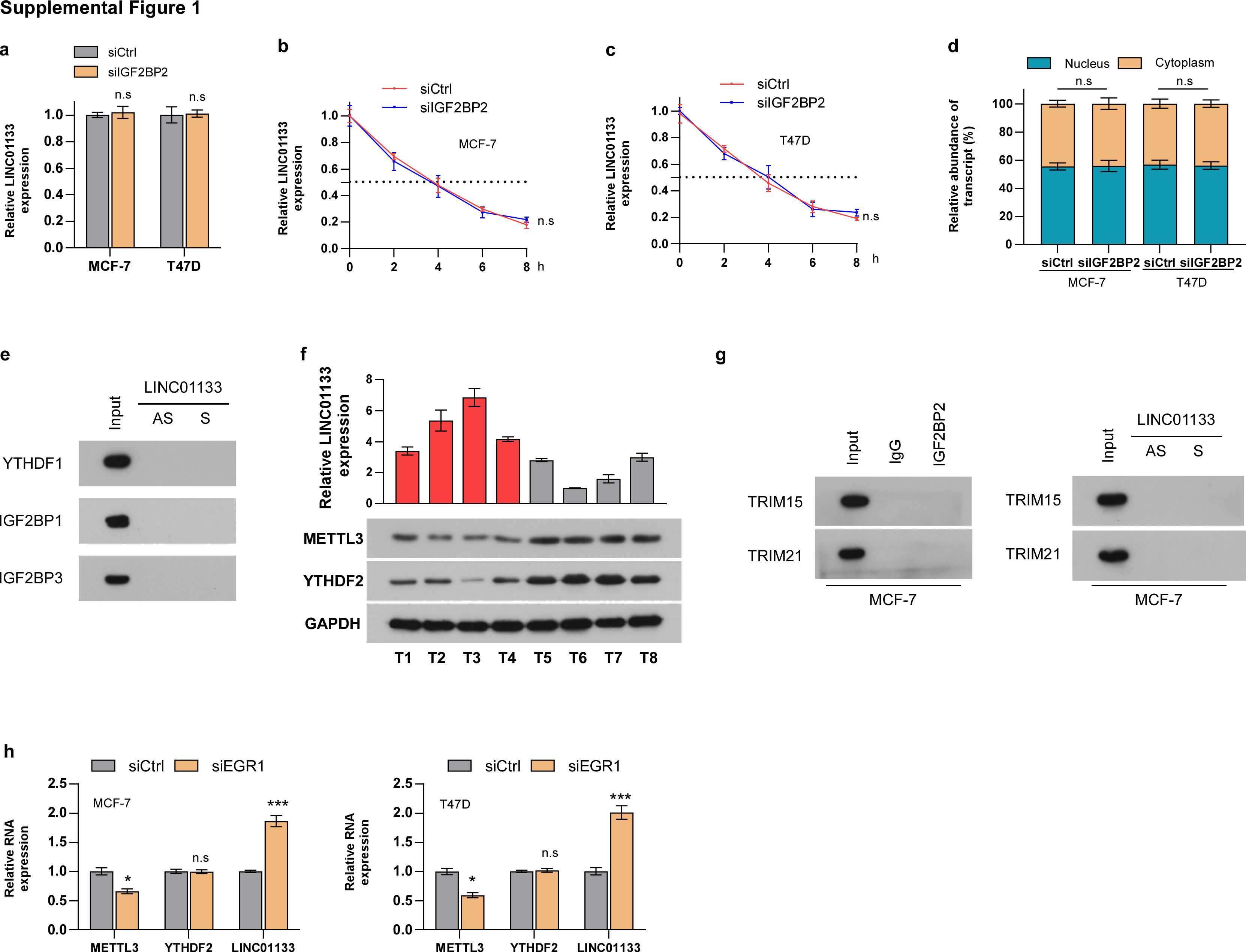

Supplement: Supplementary Figure 1 — (a) Knockdown of IGF2BP2 does not affect LINC01133 expression levels in ER+ breast cancer cells. (b, c) Knockdown of IGF2BP2 does not affect the RNA stability of LINC01133 in ER+ breast cancer cells. (d) Knockdown of IGF2BP2 does not affect subcellular location of LINC01133 in ER+ breast cancer cells. (e) IGF2BP1/3 cannot bind to LINC01133 in ER+ breast cancer cells. (f) A negative correlation exists between the protein levels of METTL3/YTHDF2 and LINC01133 expression using ER+ breast cancer tissues. (g) TRIM21/15 cannot interact with IGF2BP2 and LINC01133 in ER+ breast cancer cells. (h) Knockdown of EGR1 significantly reduces METTL3 expression while upregulating LINC01133 levels in ER+ breast cancer cells, while YTHDF2 remains unaffected by EGR1 depletion. [file Image1.tif]
