# Supplementary material for: The m6A modification of LINC01133 suppresses ER+ breast cancer progression by modulating IGF2BP2 protein stability via a ubiquitination-dependent mechanism
Source: Front Oncol. 2025 Jun 26;15:1608574. doi: 10.3389/fonc.2025.1608574 (PMC12241053; doi:10.3389/fonc.2025.1608574)
Supplement: Supplementary file 2 [file Table1.docx]

**Supplementary Table 1.** Clinicopathological characteristics of ER+ breast cancer specimens.

| **Parameters** | **Number of cases (%)** | |
| --- | --- | --- |
| **Gender** |  |  |
|  | Male | 0 (0%) |
|  | Female | 88 (100%) |
| **Age(years)** |  |  |
|  | < 55 | 51 (57.95%) |
|  | ≥ 55 | 37 (42.05%) |
| **T classification** |  |  |
|  | T1 | 29 (32.95%) |
|  | T2 | 45 (51.14%) |
|  | T3 | 10 (11.36) |
|  | T4 | 4 (4.55%) |
| **N classification** |  |  |
|  | LN- | 31 (35.23%) |
|  | LN+ | 57 (64.77%) |
| **Clinical stage** |  |  |
|  | Ⅰ | 11 (12.50%) |
|  | Ⅱ | 43 (48.86%) |
|  | Ⅲ | 34 (38.64%) |
| **ER status** |  |  |
|  | Negative | 0 (0%) |
|  | Positive | 88 (100%) |
